# Nkx2.2+ Progenitors Generate Somatic Motoneurons in the Chick Spinal Cord

**DOI:** 10.1371/journal.pone.0051581

**Published:** 2012-12-17

**Authors:** Hitoshi Gotoh, Katsuhiko Ono, Tadashi Nomura, Hirohide Takebayashi, Hidekiyo Harada, Harukazu Nakamura, Kazuhiro Ikenaka

**Affiliations:** 1 Department of Biology, Kyoto Prefectural University of Medicine, Kyoto, Japan; 2 Division of Neurobiology and Anatomy, Graduate School of Medical and Dental Sciences, Niigata University, Niigata, Japan; 3 Japan Science and Technology Agency (JST), PRESTO, Saitama, Japan; 4 Department of Molecular Neurobiology, Graduate School of Life Sciences, Institute of Development, Aging and Cancer, Tohoku University, Miyagi, Japan; 5 Division of Neurobiology and Bioinformatics, National Institute for Physiological Sciences, Aichi, Japan; 6 Department of Physiological Sciences, School of Life Science, The Graduate University of Advanced Studies (Sokendai), Kanagawa, Japan; Universitat Pompeu Fabra, Spain

## Abstract

Heterogeneous classes of neurons are present in the spinal cord and are essential for its function. Expression patterns of transcription factors in neural progenitor cells determine neuron subtypes during development. Nkx2.2 is expressed in the progenitor cell pool located just ventrally to the Olig2-positive pool and is indispensable for V3 interneuron generation in the spinal cord and also for visceral motoneuron generation in the hindbrain. However, whether Nkx2.2-positive progenitor cells generate diverse classes of neuron is not fully understood. Using a chick lineage tracing method in a genetically-defined manner, we found that Nkx2.2-expressing progenitor cells differentiate into general visceral motoneurons as well as *sim1*-positive V3 interneurons. Surprisingly, we further observed that Nkx2.2-expressing progenitors differentiate into somatic motoneuron. Our findings suggest that the different classes of motoneurons are derived from more complex sources than were previously expected in the chick spinal cord.

## Introduction

The spinal motor circuitry that generates motor output consists of several types of motoneurons and interneurons. It is well known that the motoneuron forms a columnar structure along the rostro-caudal axis and each columnar neuron shows distinct muscle innervation patterns in both chicks and mice [1]. At limb levels, motoneurons form the medial motor column (MMC) which regulates trunk muscles and the lateral motor column (LMC) which regulates limb muscles, whereas the sympathetic preganglionic motor column (called the Column of Terni; CT in chicks) and MMC are formed in the thoracic spinal cord.

A gradient of morphogens, such as sonic hedgehog, induces expression of transcription factors that specify different types of neurons as well as glial cells. Progenitor cells of somatic motoneurons are located in the ventral neural tube and express Olig2, a basic helix-loop-helix transcription factor, thus forming the pMN domain. Olig2 initially specifies motoneurons and genetic deletion of *olig2* causes loss of motoneurons in mice [2]–[4]. Nkx2.2 is a homeodomain transcription factor and is expressed just ventrally to the pMN domain, demarcating the p3 domain, and is required for V3 interneuron development [5]. These transcription factors show cross-repressive interactions [6], suggesting that their functional interaction is important for formation of the boundary between pMN and p3 domains. By using an in ovo electroporation technique, it was shown that forced expression of Nkx2.2 represses expression of Olig2 [7], thus Nkx2.2 itself is considered to be a negative regulator of motoneuron generation [8]. However, in the mouse hindbrain, Nkx2.2 positive cells differentiate into visceral motoneurons as well as serotonergic neurons [9]. Moreover, it was suggested that Nkx2.2-lineage cells contribute to visceral motoneurons in the spinal cord [10]. These reports raised the possibility that various types of neurons were generated from Nkx2.2 positive progenitors. However, whether a population of motoneurons derives from Nkx2.2-expressing progenitors is not fully understood in the chick spinal cord.

Here, we analyzed cell lineage from Nkx2.2-positive progenitors using the genetically-defined lineage tracing method in the chick spinal cord, which we developed recently [11]. In addition, we applied a new strategy for lineage tracing by electroporating floxed reporter plasmids and Cre expressing plasmids at quite low concentrations determined by limiting dilutions. The results show that Nkx2.2-expressing cells generate not only *sim1*-expressing V3 interneurons but also visceral motoneurons. Surprisingly, these progenitors also differentiate into somatic motoneurons in the spinal cord. Our results indicate that Nkx2.2-progenitor cells produce a highly diverse population of motoneurons as well as V3 interneurons in the chick spinal cord.

## Materials and Methods

### Animals and Gene Manipulation

Fertilized white leghorn eggs were obtained from the Ghen Corporation (Gifu, Japan) or the Yamagishi Corporation (Mie, Japan) and were incubated at 38°C. Embryonic stages of chicks were determined according to Hamburger and Hamilton [12]. All experimental procedures were approved by the Animal Care Committee of the National Institute for Physiological Sciences and that of Kyoto Prefectural University of Medicine (No. M21–162).

### In Ovo Lineage Tracing Analysis

Approximately 0.1 µl of the mixed solution containing 400 ng/µl of pNkx2.2-mCAT1-*myc* [11] and a 1/10 volume of 0.5% fast green was injected into the neural tube of HH stage 13 to 14 chicken embryos. Needle type electrodes were placed near the lumbar neural tube of the embryo and a 20 V, 30 ms pulse was applied three times using an electronic stimulator (SEN-3310; Nihon Kohden, Japan). For the retroviral injection, approximately 0.1 µl of virus solution (titer of retrovirus was 1×10^9^ cfu/ml) was injected into the neural tube 24 h after electroporation. EGFP-expressing high-titer retroviral particles were prepared and concentrated as previously described [13]. For Cre-loxP lineage tracing, mCAT1 was excised from pNkx2.2-mCAT1-*myc* and replaced by Cre (pNkx2.2-Cre). Approximately 0.1 µl of the mixed solution containing 1 ng/µl of pNkx2.2-Cre, 1 µg/µl of cAct-xstopx-nlacZ [14], and 0.05% fast green was electroporated to the neural tube. For fluorescent labeling by Cre-loxP system, the same volume of mixed solution containing 0.25 ng/µl of pNkx2.2-Cre, and 0.25 to 0.5 µg/µl of CMV-brainbow-1.0L [15] (obtained from Addgene, Boston, USA) was electroporated.

### In Situ Hybridization

Chick embryos were harvested and fixed in 4% paraformaldehyde/PBS at 4°C for 16 h, followed by incubation with DEPC-treated 20% sucrose/PBS for 24 h. For lacZ staining, chick embryos were fixed in 2% paraformaldehyde/PBS at 4°C for 1 h, followed by incubation with DEPC-treated 20% sucrose/PBS for 12 h. Embryos were embedded in OCT compound (Sakura Finetek Japan, Japan) and sections were prepared using a cryostat. Procedures of in situ hybridization were as previously described [11]. The following cDNAs were used as probes: *foxP1* (NM_001024827; nt_259-1173), retinaldehye dehydrogenas1e 2 (*raldh2*: AF181680; nt_225-1089), *sim1* (XM_419817; nt_901-1850), and *mCAT1 (slc7a1)* (Gotoh et al., 2011). Sections were observed under a microscope (BX51; Olympus, Japan).

### Immunohistochemistry

Procedures of in situ hybridization and immunohistochemistry were as previously described (Gotoh et al., 2011). For immunohistochemical staining after in situ hybridization, sections were treated with heat by microwaving for 5 min in 10 mM citrate buffer (pH 6.0) and were cooled to room temperature before incubation with primary antibodies. The primary antibodies used in this study were as follows; mouse anti-HB9, mouse anti-Nkx2.2, mouse anti-Lim3 (DSHB, University of Iowa, USA), rabbit anti-GFP (Invitrogen, USA), rabbit anti-Olig2, goat anti-ChAT (Millipore, USA), chiken anti-LacZ (Abcam, USA), and rabbit anti-Myc (MBL, Japan). Sections were observed under a fluorescent microscope (BX51; Olympus, Japan) or confocal microscope (FV-1000; Olympus, Japan).

### LacZ Staining

For lacZ staining, chick embryos were fixed in 2% paraformaldehyde/PBS at 4°C for 1 h, followed by incubation with DEPC-treated 20% sucrose/PBS for 12 h. Embryos were embedded in OCT compound (Sakura Finetek Japan, Japan) and sections were prepared using a cryostat. LacZ staining was performed as previously described [16]. For lacZ/immunohistochemistry or in situ hybridization double staining, lacZ-stained sections were fixed in 4% paraformaldehyde/PBS at room temperature for 30 min, followed by staining procedures as described above.

### Quantitative Analysis

For quantitative analysis, at least three independent experiments were performed. Sections were collected approximately every 300 µm and all sections that were positive for GFP or lacZ were counted. All quantitative data are shown as mean±SEM.

### Retrograde Labeling of Motoneurons

Twenty- four hours after the electroporation, up to 1 µl of fluorogold (FG) solution (4% solution in water; Invitrogen) was injected into wing bud of the chick embryos using a pulled glass capillary. Two days after FG injection, embryos were fixed with 4% paraformaldehyde/PBS as above-mentioned. After X-gal staining or GFP immunostaining, recombined cells were examined whether they were labeled with FG.

## Results

### Somatomotor Neuron Generation from Nkx2.2+ Progenitors at HH 19

**Figure 1:**
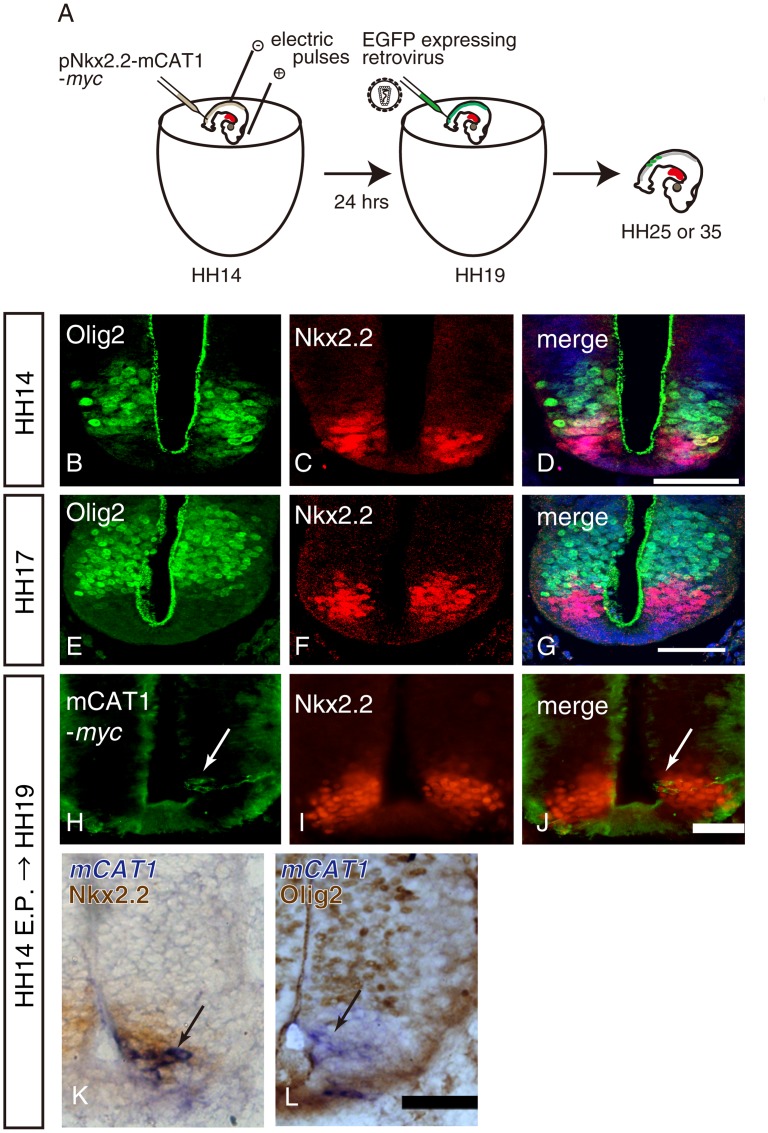
Expression of the murine retroviral receptor is specific to Nkx2.2-positive progenitors. A, A schematic diagram of the lineage tracing method of Nkx2.2-positive progenitors. It consists of the electroporation of the retroviral receptor followed by infection by the murine retrovirus. B–G, Double staining of spinal cord sections with anti-Olig2 and anti-Nkx2.2 antibodies at HH 14 (B–D) and HH 17 (E–G). H–J, Specific expression of mCAT1-*myc* in the p3 domain. pNkx2.2-mCAT1-*myc* was introduced by in ovo electroporation at HH 14, and 24 h after the electroporation, the spinal cord sections were immunostained using Myc (H, arrow) and Nkx2.2 antibodies (I). A merged image of H and I was shown in J. K and L, Expression of *mCAT1* mRNA was shown by in situ hybridization (K and L; purple, arrows) followed by immunohistochemistry using Nkx2.2 (K; brown) or Olig2 (L; brown). Scale bars indicate 50 µm.

In our previous study [11], we showed that Nkx2.2-expressing progenitor cells in the gliogenic phase differentiate into mature oligodendrocytes in the chick spinal cord. To analyze whether Nkx2.2-lineage cells generate diverse classes of neurons in the chick spinal cord, we employed the same strategy (Fig. 1A). First, the expression pattern of Olig2 and Nkx2.2 was examined within the ventricular zone. Three thoracic sections were analyzed in each embryo and four embryos were used for this analysis. In the early embryonic stage (HH stage 14), a small population of Nkx2.2/Olig2 double-positive cells were present only at the boundary of p3 and pMN domains (16.5%±1.64, percentage of Nkx2.2/Olig2 positive cells/total Nkx2.2 positive cells, n = 4; Fig. 1B-D) and double-positive cells decreased in number at HH 17 (4.14%±0.69, n = 4; Fig. 1E–G), indicating that the border became sharper as embryos developed. pNkx2.2-mCAT1-*myc*, which express mCAT1 (receptor for murine retrovirus) under the regulation of the enhancer region of *nkx2.2* [17], was introduced into the chick embryonic neural tube at HH 14 by electroporation. mCAT1-Myc was expressed exclusively in Nkx2.2-expressing cells 24 hrs after electroporation (HH 17-19; Fig. 1H-J; n = 4) as previously demonstrated in later stages [11]. This was also confirmed by in situ hybridization that showed *mCAT1* mRNA was present in Nkx2.2-expressing cells, but not in Olig2-expressing cells, just dorsally to the p3 domain within the ventricular zone (Fig. 1K and L). These observations suggest that electroporated mCAT1 was expressed only in the Nkx2.2-expressing cells in our experimental condition, thus the reliability of this system is also confined to neurogenic stages.

**Figure 2:**
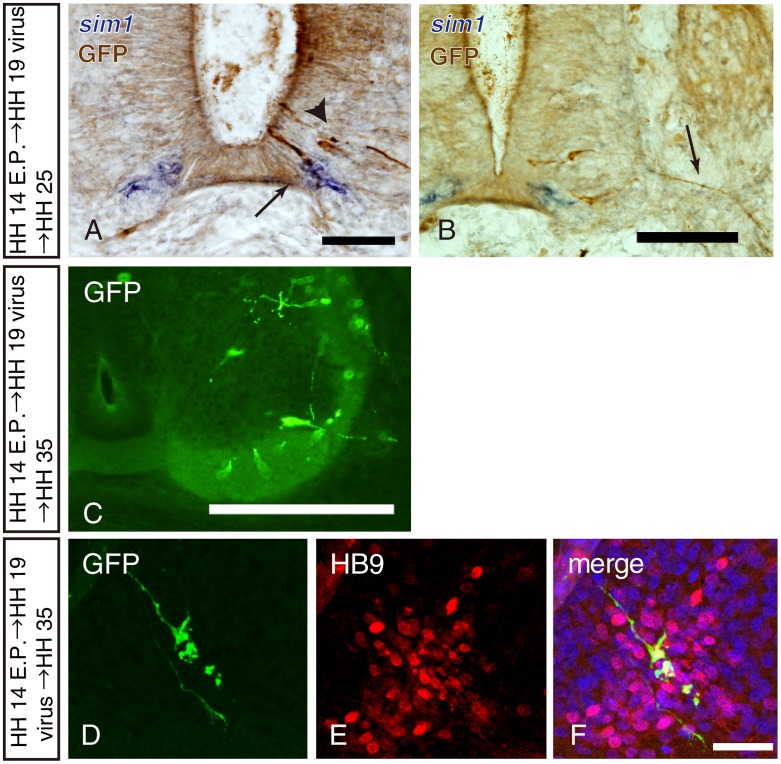
Both V3 interneurons and somatic motoneurons are generated from Nkx2.2-positive progenitors. A and B, *sim1* in situ hybridization (purple) followed by GFP immunohistochemistry (brown). Recombined cells were *sim1*-positive (arrow in A) or *sim1*-negatve (arrowhead in A). An arrow in B indicates recombined cell axon outside the spinal cord, suggesting it was a motoneuron axon. C, GFP-positive recombined cells in the HH35 spinal cord, showing a GFP-positive axon extending outside the spinal cord. D-F, Double staining with HB9 and GFP immunohistochemistry, demonstrating a HB9-positive recombind cell. Scale bars in A and B = 50 µm; in C = 200 µm; in F = 20 µm.

To trace the lineage of p3 domain cells, an EGFP-expressing retroviral solution was injected into the neural tube 24 h after electroporation of pNkx2.2-mCAT1-*myc* (HH 19). We analyzed embryos 24 h after retroviral transduction and found that EGFP positive cells were present in the ventral neural tube (Fig. 2A; brown). EGFP-positive cells that were observed in the most ventral area expressed *sim1* mRNA, which is a marker for V3 interneurons (Fig. 2A; arrow).

EGFP-positive cells were also observed dorsally to *sim1*-positive cells (Fig. 2A; arrow heads). Approximately 10% of total EGFP-positive cells extended their axons into the ventral roots at HH 25 (E4; Fig. 2B), one day after retroviral labeling, when most labeled cells were radially migrating cells and still located in the ventricular zone [18]. GFP-positive axons were also observed in the ventral root at HH 35 (E9; Fig. 2C). In addition, a small number of EGFP-positive neurons expressed HB9 at HH 35 in the chick spinal cord (E9; Fig. 2D–F). These data suggest that some progenitor cells in the p3 domain differentiate into HB9 positive somatic motoneurons.

### Diverse Motoneuron Generation from Nkx2.2+ Progenitors at HH14

**Figure 3:**
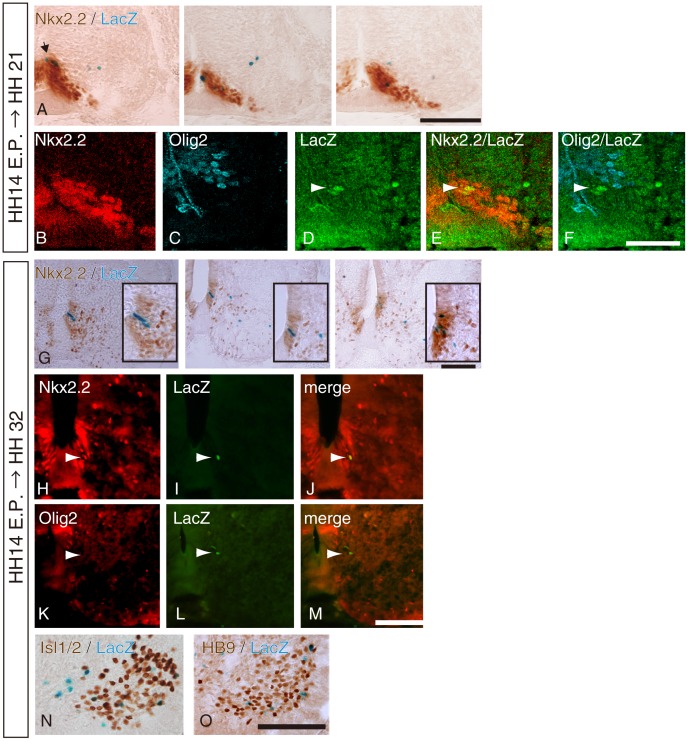
LacZ-labeled cells by electroporation with pNkx2.2-Cre;cAct-xStopx-nLacZ strongly express Nkx2.2 in the ventricular zone. pNkx2.2-Cre and cAct-xStopx-nLacZ were electroporated at HH 14 in the chick neural tube and an embryo was grown to be HH 21 (E3.5; A–F) or HH 32 (E7; G–O). A, LacZ-staining followed by immunohistochemistry using Nkx2.2 antibody. Sections were arranged in rostral (left) to caudal (right) order. B-F, Triple immunostaining with anti-Nkx2.2, anti-Olig2, and anti-LacZ antibodies. Arrowheads indicate recombined cells. G, LacZ-staining followed by immunohistochemistry using Nkx2.2 antibody. Sections were arranged in rostral (left) to caudal (right) order. Insets show higher magnification pictures of LacZ-positive cells in the Nkx2.2-positive ventricular zone. H-M, Sections at HH 32 were double-immunolabeled using anti-LacZ and anti-Nkx2.2 (H-J), or anti-LacZ and anti-Olig2 (K-M), respectively. Arrowheads indicate recombined cells. N, LacZ-positive cells with Islet1/2 immunoreactivity. O, LacZ-positive cells with HB9 immunoreactivity. Scale bars in A, G, M, O = 100 µm; in F = 50 µm.

We next analyzed the distribution of Nkx2.2-lineage motoneurons, and their contribution to the columnar structure. Because motoneuron generation in LMC or CT starts around HH 15 [19], it is conceivable that labeling by a retrovirus (HH 17 to 19) could not label all the progenitors that generate motoneurons. In addition, labeled cells by retroviral injection at HH 19 rarely differentiate into CT cells located near the central canal. In order to avoid this bias, we employed a Cre-loxP mediated lineage-tracing method; a floxed-nlacZ reporter plasmid was co-electroporated with the pNkx2.2-Cre plasmid, which is regulated by the same *nkx2.2*-enhancer, into HH 14 chick spinal cords. The minimum concentration of the pNkx2.2-Cre plasmid was determined by limiting dilutions to avoid nonspecific labeling. After 36 h, the initial LacZ expression in the ventricular zone was restricted to the Nkx2.2-positive cells at HH 21 (E3.5, Fig. 3A), which was similar to that observed in retroviral labeling. As some LacZ-positive cells were located close to Olig2-positive cells in the ventricular zone, adjacent sections were triple labeled using anti-LacZ, anti-Nkx2.2, and anti-Olig2 antibodies. Some LacZ-positive cells were present at the domain boundary and few LacZ-positive cells were observed in the Olig2+/Nkx2.2- ventricular zone whereas most cells were located in Nkx2.2+/Olig2- region (Fig.3 B-F). We counted cells in the Olig2/Nkx2.2 boundary and 13.3±8.16% (mean±SEM; n = 4) of total labeled cells within the ventricular zone cells were located at the domain boundary. In later stages (HH 32 or E7), LacZ-positive cells were observed in both the ventricular zone and ventral horn (Fig. 3L). LacZ-positive cells within the ventricular zone expressed Nkx2.2 (Fig. 3 G; inset and H-J) but not Olig2 (Fig.3 K–M). At this stages, no LacZ-positive cells expressed Olig2 at detectable levels, whereas they strongly expressed Nkx2.2 in all embryos (n = 4). Some LacZ-positive cells in the ventral horn were Isl1/2-positive and HB9-positive (Fig. 3N, O), indicating that they differentiated into somatic motoneurons.

**Figure 4:**
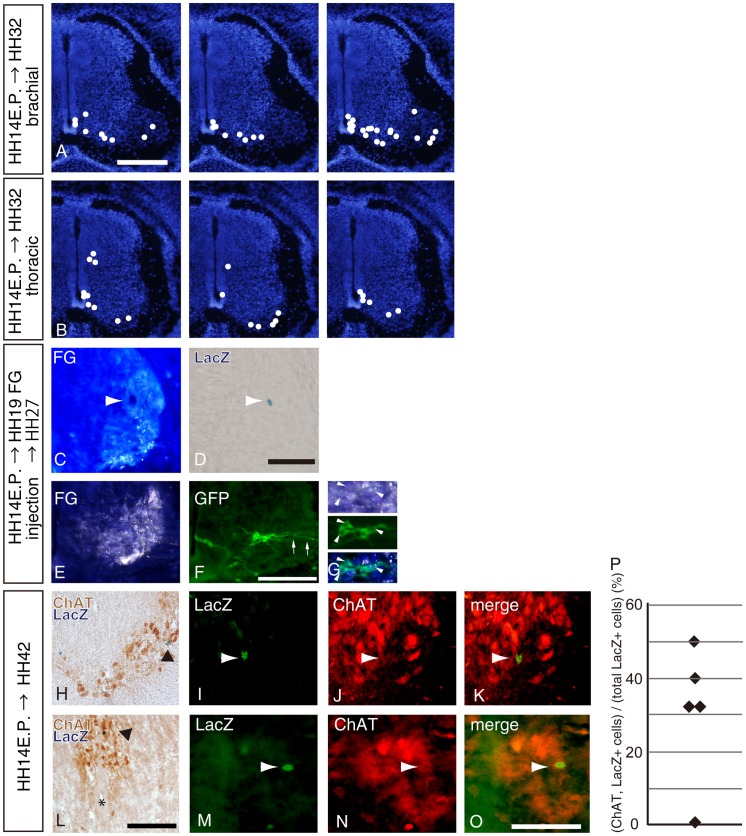
Distribution of Nkx2.2 lineage motoneurons in the spinal cord. pNkx2.2-Cre and cAct-xStopx-nLacZ were electroporated at HH 14 in the chick neural tube. A and B, LacZ-positive cells of one chick spinal cord at HH 32 (E7) were superimposed and marked with dots on a schematic diagram. Three independent experiments were performed in the brachial spinal cord (A) or thoracic spinal cord (B). C and D, A LacZ-positive cell in the ventral horn in which most of the motoneurons were retrogradely labeled with fluorogold (FG). E–G, GFP-positive recombined cells with brainbow system in the ventral horn were retrogradely labeled with FG (G; arrowheads). GFP-positive axons were extending toward the ventral root (arrows). H and L, Sections of HH 42 spinal cord were subjected to LacZ-staining followed by immunohistochemistry using ChAT antibody. LacZ-positive cells in the ventral horn (H; somatomotor neuron) and close to the central canal (L; preganglionic CT neuron) are shown. Arrowheads indicate double positive cells and the asterisk shows the location of the central canal. I–K and M–O, Expression of ChAT in LacZ-positive cells was also analyzed by double fluorescent labeling (I–K, M–O). P, LacZ/ChAT-positive cells of one chick spinal cord were counted and divided by the total number of LacZ-positive cells and were shown as percentages (P). Five chick embryos were examined in this study. Scale bars in A and L = 200 µm; D, F and O = 50 µm.

To define the distribution of Nkx2.2-lineage cells, we stained brachial or thoracic spinal cord sections by LacZ histochemistry. Fig. 4A and 4B show schematic diagrams of the distribution of LacZ-positive cells in the brachial or thoracic spinal cord of three independent chick embryos. At HH 32 (E7), LacZ-positive cells were mostly present in the gray matter and the ventricular zone. In two of three embryos (Fig. 4A, left and right), there were LacZ-positive cells in the ventral horn in the brachial spinal cord. In the thoracic spinal cord, LacZ-positive cells were observed more dorsally near the ventricular zone, suggesting that they were preganglionic motoneurons (Fig. 4B). In order to confirm that recombined cells included motoneurons that send axons outside the spinal cord, retrograde tracer (fluorogold; FG) was used to label motoneurons. Injected FG was spread not only to the wing bud but also widely to the body cavity including peritoneal and thoracic ones, and therefore, somites and other mesenchymal tissues showed FG fluorescence bilaterally in the coronal sections. Within the spinal cord, somatic motoneurons in the ventral horn and preganglionic cells in the intermediate region showed yellowish white FG fluorescence bilaterally. In cases with FG injection, a LacZ-positive cell was located in the ventral horn where most of cells were retrogradely labeled with FG so that this LacZ-positive cells was highly likely labeled with FG also (Fig. 4C, D). To further analyze this, we used the brainbow plasmid which expresses RFP, and GFP after Cre mediated recombination. GFP-positive cells observed in the ventral part of the ventral horn showed GFP immunoreactivity, one of which was retrogradely labeled with FG (Fig.4E, F, and G arrowheads). Therefore, some of the recombined cells were apparently motoneurons that sent axons outside the spinal cord (Fig. 4F arrows). At later stages (HH 42 or E16), some LacZ-positive cells expressed choline acetyltransferase (ChAT), an enzyme for acetylcholine generation and, thus, often used as a marker for mature motoneurons, in the ventral horn and around the central canal (Fig. 4H and 4L). Expression of ChAT in recombined cells were also confirmed by double-immunohistochemistry using anti-LacZ and anti-ChAT antibodies (Fig. 4I–K, 4M–O; arrowheads). We collected 20 µm sections in each 300 µm section from electroporated spinal cord. The ratio of LacZ/ChAT-double positive cells per total LacZ-positve cells was calculated from five embryos. A large number of motoneurons were derived from Nkx2.2-positive cells (Fig. 4P; average = 31.3±8.4%). ChAT-negative cells might represent interneuron other than motoneurons. These data suggest that a large population of Nkx2.2-progenitors differentiated into functional motoneurons.

**Figure 5:**
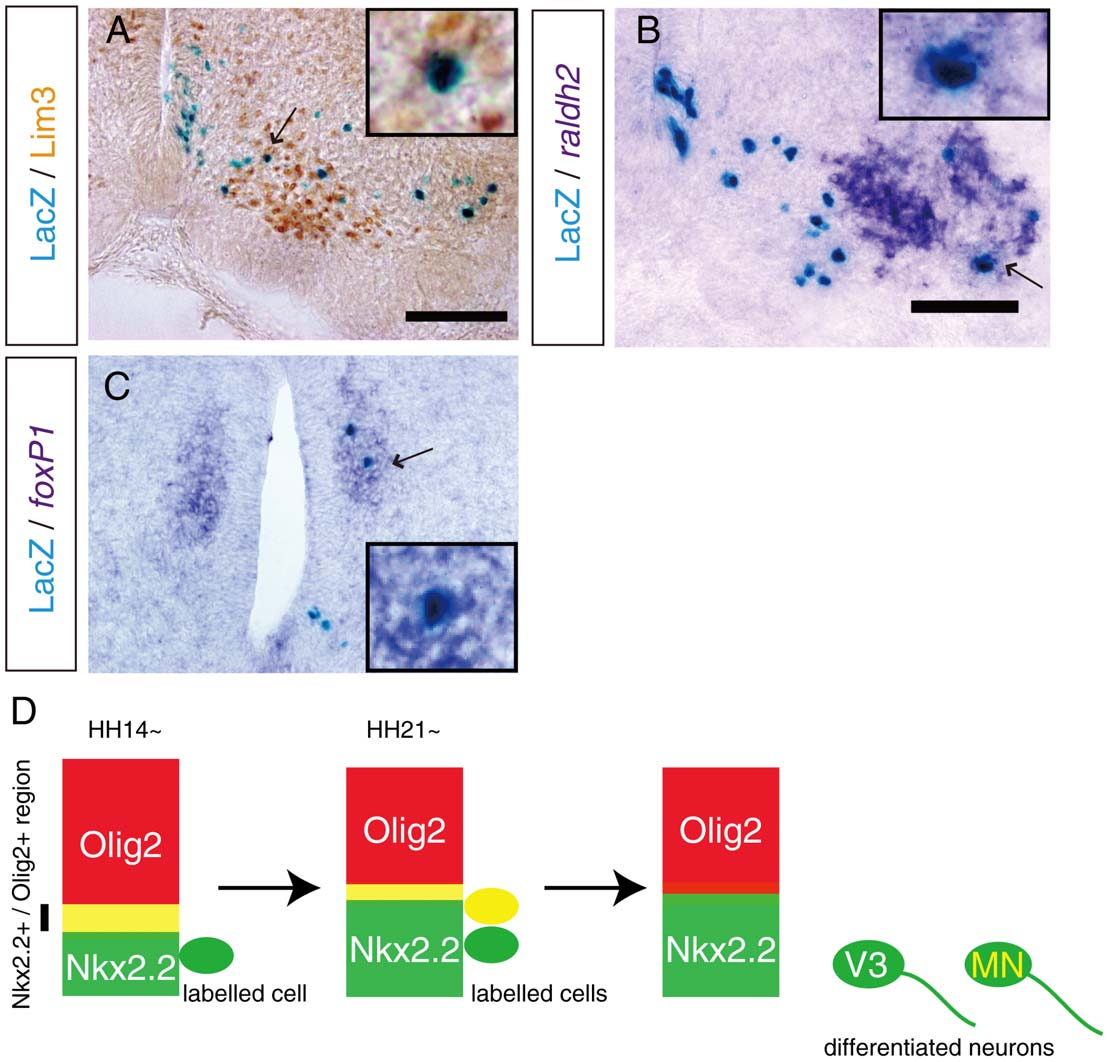
Nkx2.2-positive progenitors contribute to all types of motoneurons. pNkx2.2-Cre and cAct-xStopx-nLacZ were electroporated at HH 14 in the chick neural tube and an embryo was grown to be HH 32 (E7). A-C, Sections were subjected to LacZ-staining followed by immunohistochemistry using Lim3 antibody for MMC neurons (A), in situ hybridization for *raldh2* for LMC neurons (B), or *foxP1* for CT neurons (C), respectively. Arrow indicates double positive cells and magnified images of double positive cells are shown in the inset of each figure. D, A schematic representation of our hypothetical mechanism for motoneuron generation from Nkx2.2-positive progenitors. Scale bars indicate 100 µm.

In order to define the motoneuron subtype from Nkx2.2-positive progenitors, we stained LacZ together with subtype-specific markers [20], [21] at HH 32 in brachial or thoracic spinal cords using Lim3 for MMC, *raldh2* for LMC, and *foxP1* for CT. Nkx2.2-derived cells differentiated into Lim3 positive (Fig. 5A) and *raldh2*-positive motoneurons (Fig. 5B) in the brachial spinal cord. In the thoracic spinal cord, we observed LacZ/Lim3 double positive MMC (data not shown) as well as LacZ/*foxP1* double positive preganglionic neurons (Fig. 5C). These observations suggest that Nkx2.2-positive progenitors differentiate into all subtypes of motoneurons.

## Discussion

In this study, we used genetically-defined lineage tracing analysis for Nkx2.2-expressing progenitors using a murine retrovirus [11]. Furthermore, we applied a new electroporation of Cre-loxP mediated lineage tracing strategy and showed the relevance of this strategy. We used Cre at the concentration of end-point dilution, together with floxed lacZ plasmids. Initial LacZ-positive cells that were recombined by Nkx2.2-Cre were always positioned in the Nkx2.2-positive cell population, but not in Olig2+/Nkx2.2- ventricular zone cells (Fig. 3). In addition, LacZ-positive cells were not observed when the Cre-driver plasmid was diluted to 0.1 ng/µl (1/10 dilution of our labeling condition). Finally, the distribution of LacZ-positive cells was similar to that of retroviral labeling. These observations support the relevance of our Cre-loxP labeling strategy with limiting dilutions of the Cre plasmid.

It has been reported that overexpression of Nkx2.2 repress the expression of Olig2 or HB9 [7], [22], [23], suggesting that Nkx2.2 has a negative effect on motoneuron lineage. However, whether there is a direct lineage relationship between Nkx2.2-positive progenitors and motoneurons remains to be resolved due to the lack of lineage tracing analysis from Nkx2.2-positive progenitors. We used the above mentioned methods for analyzing Nkx2.2-lineage cells in the chick spinal cord. Our finding first shows that Nkx2.2-positive progenitor cells in chick spinal cords generate not only V3 interneurons but also Terni column cells (Fig. 4), the avian visceral preganglionic motoneurons located near the central canal. In the mouse hindbrain, Nkx2.2 has been reported to be expressed in progenitor cells that give rise to both visceral motoneurons and serotonergic neurons [9], which supports our observation that Nkx2.2-positive progenitors contributed to different classes of neurons in the chick spinal cord. Surprisingly, we found that Nkx2.2-positive progenitors also gave rise to somatic motoneurons. It was reported that preganglionic motoneurons have a close lineage relationship with somatic motoneurons as demonstrated by the tritium thymidine labeling method [24]. It is well-known that motoneurons are arranged into columnar structures and their arrangement is dependent on the combinatorial expression of transcription factors [1], [25], thus we analyzed the subtype of Nkx2.2-lineage motoneurons. Since a recent report suggested that the pMN domain can be subdivided into a ventral part and a dorsal part and that the ventral part of the pMN domain contributes to MMC motoneurons [26] and cells derived from Nkx2.2-positive progenitors were present in the most dorsal part of the p3 domain, it is possible that the dorsal part of the p3 domain would contribute to MMC motoneurons. However, we found that Nkx2.2-positive progenitors gave rise to preganglionic neurons as well as somatic motoneurons in LMC and MMC within the ventral horn (see Fig. 4), thus no preferential differentiation into MMC motoneurons was observed, suggesting different regulatory mechanisms of motoneurons development from Nkx2.2-positive progenitors. Although clonal analysis would help us to analyze whether GFP-labeled motoneurons and V3 interneurons are derived from the same progenitor cells, it is difficult to perform clonal analysis using our systems because expression levels of mCAT1 are dependent on the concentration of plasmids or the electroporated area in the spinal cord as previously described [11].

The molecular mechanisms which regulate motoneuron development from the p3 domain are unknown whereas Nkx2.2 is reported to have inhibitory functions in motoneuron development [8]. It is known that Olig2 is essential for motoneuron development as revealed by a gain-of-function study using chick embryos [7] as well as loss-of –function studies using *olig2*-deficient mice [2]–[4]. We observed that LacZ-positive cells were located at the pMN/p3 domain boundary with strong Nkx2.2 expression. Although initialy mCAT1 or LacZ-positive cells in the ventricular zone were restricted to the p3 (Nkx2.2-positive) domain, a small population of labeled cells was present at the Olig2/Nkx2.2 boundary. Our data showed that 13.3% of the labeled cells were present at the domain boundary at HH21 (E3.5) and no labeled cell was located at Olig2-positive area at HH32 (E7). The percentage of mature ChAT-positive neurons to ChAT-negative neurons (31.3%) at more later stage (HH 42, E16) suggest that early Nkx2.2 progenitors in the domain boundary might express Olig2 transiently in a short time window and differentiate into motoneurons (Fig. 5D). Using labeling techniques including retroviral clonal analysis, it was reported that chick neural progenitor cells show extensive migration along the dorso-ventral axis inside the ventricular zone [27]–[29]. These reports raised the possibility that p3 progenitor cells acquire Olig2 expression during the dorso-ventral migration of progenitor cells, and then differentiate into motoneurons. It was suggested that motoneurons have a lineage relationship with oligodendrocytes with respect to the requirement of Olig2 [16]. Mouse oligodendrocytes are generated from Olig2-positive cells during glial cell development, whereas oligodendrocytes are developed from Nkx2.2-positive p3 domain cells in the chick spinal cord [11], [30], [31]. Therefore, there may be subtle species differences in the developmental origin of oligodendrocytes [32] as well as motoneurons in the chick spinal cord, as suggested by the present study. It is interesting to examine whether or not Nkx2.2-lineage cells differentiate into motoneurons in the mouse spinal cord.

In conclusion, we found that p3 domain progenitor cells contributed to all kinds of motoneuron subtypes in the chick spinal cord, suggesting that diverse embryonic origins contribute to diverse mature motoneurons.
